# PROTAC‐Engineered Protein/DNA Nanoantigen is a Potent Booster for Dendritic Cell Vaccines in Cancer Immunotherapy

**DOI:** 10.1002/advs.77089

**Published:** 2026-08-11

**Authors:** Peng Liu, Qiang Chen, Zhe Zhou, Yuekang Xu, Jinyao Li

**Affiliations:** ^1^ Xinjiang Key Laboratory of Biological Resources and Genetic Engineering College of Life Science and Technology Xinjiang University Urumqi China

**Keywords:** cancer immunotherapy, dendritic cell vaccine, protein/DNA‐integrated nanoantigen platform, proteolysis‐targeting chimera

## Abstract

The efficacy of dendritic cell (DC) vaccines in cancer immunotherapy is often limited by weak immunogenicity due to inefficient antigen cross‐presentation (XPT). We previously combined a calcium carbonate (CaCO_3_) delivery system with a proteolysis‐targeting chimera (PROTAC) to enhance antigen degradation. Along this line, we developed a protein/DNA‐integrated nanoantigen platform (HpOAC) co‐loading CaCO_3_ with a His‐tagged ovalbumin (O) modified with the von Hippel‐Lindau (VHL) E3 ligase‐recruiting peptide ALAPYIP (HOA) and a eukaryotic plasmid encoding O‐ALAPYIP (pOA). HpOAC promoted DC maturation and migration via Ca^2+^ release. Mechanistically, HOA provided an immediate antigen source, while pOA enabled sustained expression; both underwent ALAPYIP‐mediated VHL recruitment, enhancing O ubiquitination and proteasomal degradation, thereby amplifying XPT. Consequently, HpOAC‐DCs induced strong OT‐I/OT‐II T cell proliferation in vitro. In vivo, a single immunization with HpOAC‐DCs significantly suppressed tumor growth and elicited durable antigen‐specific T helper type 1 and cytotoxic T lymphocyte responses, outperforming single‐antigen vaccines. Under a two‐dose regimen, its anti‐tumor efficacy was improved compared to non‐PROTAC and in situ antigen vaccines. Thus, we constructed a PROTAC‐enhanced, protein/DNA‐integrated DC vaccine platform that synergizes immediate and sustained antigen supply with Ca^2+^‐mediated adjuvanticity, offering a highly promising strategy for novel DC vaccine design.

## Graphical abstract

A PROTAC‐functionalized protein/DNA‐integrated nanoantigen platform is developed to enhance dendritic cell vaccine potency. By combining a VHL‐recruiting peptide‐modified antigen with a sustained expression plasmid within CaCO_3_ nanoparticles, this system amplifies cross‐presentation, promotes DC maturation, and elicits robust Th1 and CTL responses, offering a promising strategy for cancer immunotherapy.

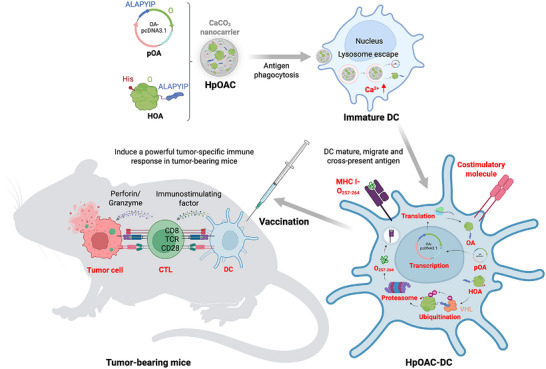

## 1 Introduction

Whether as DNA or protein vaccines, both antigenic forms need to be internalized by antigen‐presenting cells (APCs) upon administration [1, 2]. Specifically, upon transcription and translation within APCs, DNA vaccines are converted into proteins, which are subsequently degraded into antigenic peptides by cytosolic proteasomes [3]. These peptides are then directly presented via major histocompatibility complex (MHC) I, thereby activating CD8^+^ T cells. In contrast, protein vaccines are typically degraded by lysosomes in APCs and subsequently presented through the MHC II pathway, leading to the activation of CD4^+^ T cells [4]. However, the cross‐presentation (XPT) mechanism of professional APCs, such as dendritic cells (DCs), also enables protein vaccines to be presented on MHC I to CD8^+^ T cells [5]. This unique antigen presentation is achieved through a series of sequential processes involving antigen endosomal escape, degradation by the ubiquitin‐proteasome system (UPS), peptide assembly within the endoplasmic reticulum, and transport via the Golgi apparatus [6, 7]. Once activated, CD8^+^ T cells, which serve as the central effectors of anti‐tumor immune responses, differentiate more efficiently into cytotoxic T lymphocytes (CTLs) with the assistance of the CD4^+^ T helper type 1 cell subset (Th1) [8, 9, 10]. The IFN‐γ secreted by these CTLs can, in turn, promote Th1‐type immune responses, thereby establishing a positive feedback loop that enhances the overall cellular adaptive immune response [11, 12, 13].

Tumor neoantigens refer to novel proteins generated by somatic mutations in tumor cells that are recognizable by the immune system and are not expressed in normal tissues [14]. DCs, which possess the most potent XPT capacity among APCs, can elicit robust tumor‐specific immune responses when treated with the tumor neoantigens, while maintaining a favorable safety profile without significant toxicity [15, 16]. As a model antigen, ovalbumin (OVA, O) is extensively utilized in the field of tumor immunology for studying neoantigen‐like immune responses [17]. In our previous study, inspired by the mechanism of proteolysis‐targeting chimera (PROTAC), we covalently conjugated a recruiter of the E3 ubiquitin ligase cereblon (CRBN) with O and then loaded this chimeric antigen into a calcium carbonate (CaCO_3_) nanoparticle to make a superior DC vaccine that elicited robust antigen‐specific CTL and Th1‐type immune responses in B16‐OVA tumor‐bearing mice, leading to significant control of tumor progression [18]. In addition, our group utilized recombinant *Lactococcus lactis* (*L.L*) as a live vector to achieve enrichment of antigens expressed from both eukaryotic and prokaryotic systems within DCs, thereby concurrently enhancing both the direct presentation and XPT pathways, and the resulting DC vaccine exhibited superior anti‐tumor efficacy compared to that derived from recombinant *L.L* equipped with only a single expression system [19].

In this study, we aimed to persistently supply the immunogenic antigen in the super DC vaccine and enhance its efficacy in activating the Th1‐CTL positive feedback loop in tumor‐bearing mice. Specifically, we genetically fused the von Hippel‐Lindau (VHL) E3 ubiquitin ligase‐recruiting peptide ALAPYIP (A) with O to generate OA. This chimeric molecule was then produced both as a eukaryotic expression plasmid (pOA) and as a prokaryotic expression His (H)‐tagged recombinant protein (HOA), and subsequently co‐loaded into CaCO_3_ nanoparticles to obtain HpOAC. HpOAC was used to establish an integrated protein/DNA‑based DC vaccine preparation platform in vitro for cancer therapy. Importantly, we demonstrated that HpOAC‐DCs exert superior cancer immunotherapeutic effects compared to DC vaccines generated from single‐component antigens (either pOAC‐DCs or HOAC‐DCs) or from OpOC‐DCs lacking the ALAPYIP peptide. This enhanced efficacy is attributed to the PROTAC‐empowered synergy between the immediate availability of the HOA protein antigen and the sustained antigen supply from the pOA plasmid, which collectively amplifies the immunogenicity of the DC vaccine. Furthermore, direct administration of HpOAC‐antigen as an in situ vaccine in tumor‐bearing mice resulted in suboptimal anti‐tumor outcomes, likely due to the compromised phagocytosis and presentation of the antigen by the endogenous APCs in the presence of immunosuppressive tumor microenvironment.

## 2 Results

### 2.1 Preparation and Characterization of HpOAC Nanoparticles

**FIGURE 1:**
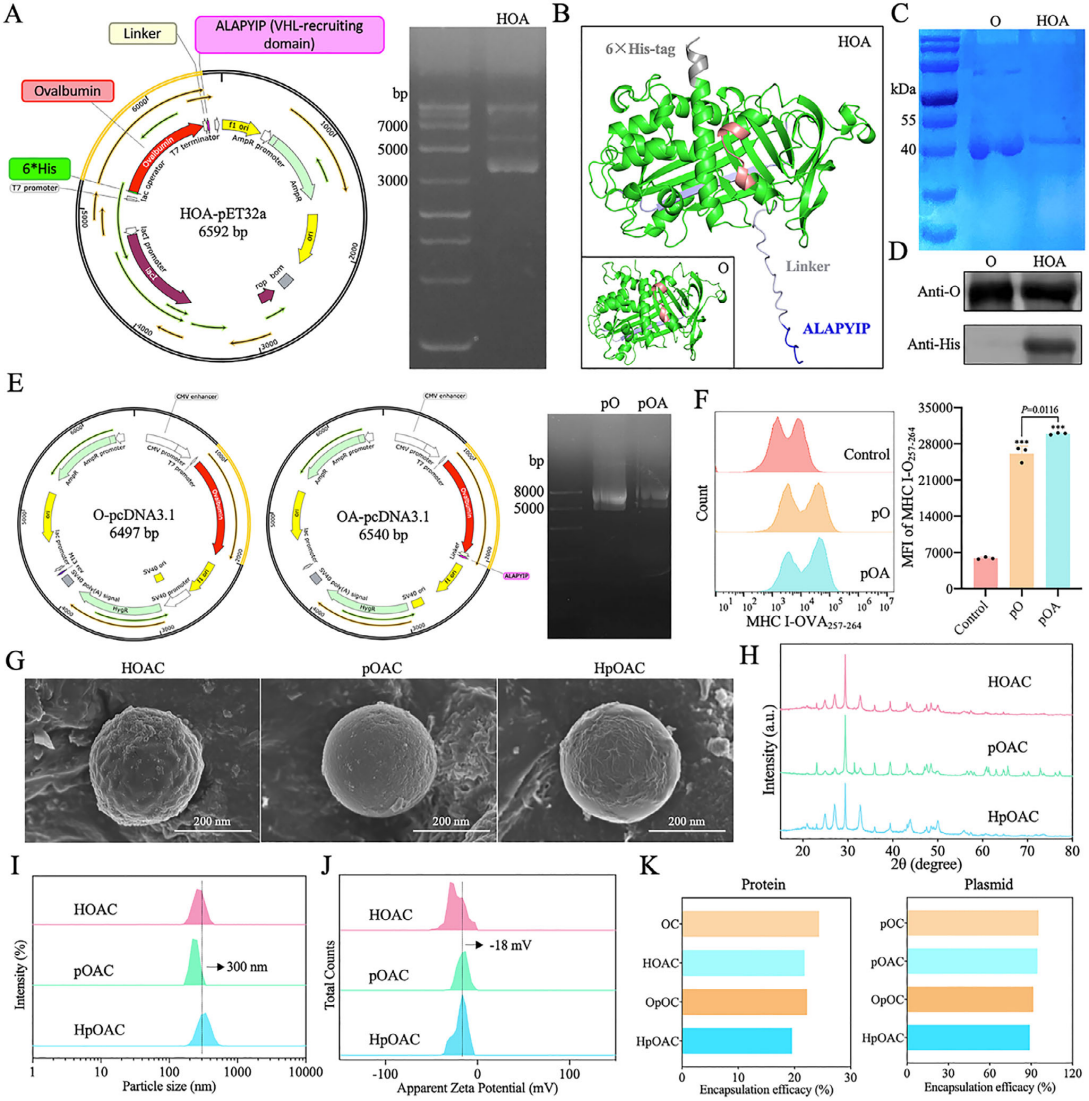
Preparation and characterization of HpOAC. (A) The prokaryotic expression plasmid map of HOA using pET32a as the vector and the corresponding agarose gel electrophoresis detection results; (B) The protein structures of HOA and O were simulated using AlphaFold; (C) The molecular weight and purity of the HOA protein were characterized by SDS‐PAGE; (D) HOA was identified by WB using antibodies against O and His‐tag; (E) The plasmid maps of pcDNA3.1‐based eukaryotic expression plasmids for O and O‐ALAPYIP and the results of agarose gel electrophoresis; (F) After transfection of DCs with pO or pOA using Lipofectamine 3000, the expression level of the MHC I‐OVA_257‐264_ complex on the DC surface was detected by FCM; (G) The morphology of HOAC, pOAC, and HpOAC was detected by scanning electron microscopy; (H) The crystal types of HOAC, pOAC, and HpOAC were determined by x‐ray diffraction; (I) Particle size distribution to reflect sizes of HOAC, pOAC, and HpOAC; (J) Zeta potential analysis to reflect HOAC, pOAC, and HpOAC dispersion system stability; (K) The protein or plasmid encapsulation efficacy of OC, HOAC, OpOC, HpOAC, pOC, and pOAC was measured. ^*^ *p* < 0.05, ^**^ *p* < 0.01, ^***^ *p* < 0.001 compared with untreated group by ANOVA. ^#^ *p* < 0.05, ^##^ *p* < 0.01, ^###^ *p* < 0.001 compared with both groups by unpaired *t*‐tests. DC: dendritic cell; O: ovalbumin; HOA: His‐O‐ALAPYIP; pO: eukaryotic expression plasmid of O with pcDNA3.1 as the vector; pOA: eukaryotic expression plasmid of O‐ALAPYIP with pcDNA3.1 as the vector; MHC I: major histocompatibility complex class I; HOAC: nanoparticle formed by loading HOA with calcium carbonate; pOAC: nanoparticle formed by loading pOA with calcium carbonate; HpOAC: nanoparticle formed by loading HOA and pOA with calcium carbonate; OC: nanoparticle formed by loading O with calcium carbonate; pOC: nanoparticle formed by loading pO with calcium carbonate; OpOC: nanoparticle formed by loading O and pO with calcium carbonate.

We first obtained the DNA sequence encoding His‐Ovalbumin‐ALAPYIP (HOA, the His‐tagged ovalbumin modified with the VHL‐recruiting peptide ALAPYIP) through gene synthesis. This sequence was then inserted into the prokaryotic expression vector pET32a, yielding the recombinant plasmid HOA‐pET32a, which was subsequently amplified in *Escherichia coli* (*E. coli*, DH5α). Agarose gel electrophoresis analysis revealed three distinct bands corresponding to different conformational isoforms of the HOA‐pET32a plasmid, representing, from bottom to top, the supercoiled, linear, and open circular forms (Figure 1A). The linear conformation was approximately 6500 bp in length, consistent with the size of 6592 bp indicated on the plasmid map. Additionally, the protein structures of O and HOA were simulated using AlphaFold. The results indicated that the incorporation of the His‐tag and ALAPYIP peptide did not substantially alter the structure of O. Moreover, both the His and ALAPYIP moieties of HOA were exposed on the protein surface, which may facilitate subsequent protein purification or contribute to their biological functions (Figure 1B). Subsequently, the HOA‐pET32a plasmid was transformed into the *E. coli* BL21 strain for protein expression. After optimizing the expression conditions (as shown in Figure S1), the HOA protein was purified using nickel affinity chromatography. The results of sodium dodecyl sulfate polyacrylamide gel electrophoresis (SDS‐PAGE) and western blot (WB) indicated that the purity of HOA exceeded 95%, its apparent molecular weight was slightly higher than that of O (Figure 1C), and it carried a His‐tag characteristic of prokaryotic expression (Figure 1D). The DNA sequences of O and OA were obtained by polymerase chain reaction (PCR) and subsequently inserted into the eukaryotic expression vector pcDNA3.1 via double enzyme digestion. The resulting constructs, O‐pcDNA3.1 (pO) and OA‐pcDNA3.1 (pOA), were verified by agarose gel electrophoresis to be approximately 6500 bp in length, consistent with the plasmid map (Figure 1E; see Table S1 for primer sequences and Figure S2 for DNA sequencing results). Importantly, 72 h after transfection of DCs with pO or pOA using Lipofectamine 3000 (WB verification is shown in Figure S3), the XPT of antigen was assessed by flow cytometry (FCM). The results showed that the level of MHC I‐O_257‐264_ was significantly higher in the pOA group compared to the pO group, demonstrating that the ALAPYIP peptide promotes the XPT of O by DCs (Figure 1F).

To promote efficient XPT, we utilized calcium carbonate (CaCO_3_) nanoparticles—which possess both antigen delivery and adjuvant effects—as a nanocarrier for the co‐delivery of HOA and pOA to DCs. Following standard procedures for morphological examination by scanning electron microscopy, the HOAC (CaCO_3_ loaded with HOA only), pOAC (CaCO_3_ loaded with pOA only), and HpOAC (CaCO_3_ loaded with both HOA and pOA) were all found to exhibit a spherical morphology (Figure 1G). The transmission electron microscopy result was consistent with that of scanning electron microscopy, showing that HpOAC exhibited a spherical morphology (Figure S4). Furthermore, x‐ray diffraction analysis confirmed that all nanoparticles possessed a vaterite‐type crystal structure (Figure 1H), which is characterized by efficient antigen delivery and favorable biocompatibility [20]. Subsequently, the particle size and zeta potential of HpOAC were analyzed using dynamic light scattering. The results showed that HOAC, pOAC, and HpOAC all exhibited an average particle size of approximately 300 nm and a zeta potential of approximately ‐18 mV, indicating that these nanoparticles are conducive to DC uptake and tend to maintain a relatively stable dispersed state in solution (Figure 1I,J). We further dispersed HpOAC in complete RPMI‐1640 medium supplemented with 10% serum and collected the nanoparticles at 0, 12, 24, and 48 h for particle size measurement (the 48 h time point included both conditions with and without DCs). The results showed that the particle size of HpOAC remained at approximately 350 nm without obvious aggregation, indicating that during co‐incubation with DCs, the CaCO_3_ nanoparticles maintained a well‐dispersed state in serum‐containing medium (Figure S5). Finally, the loading efficiencies of the different CaCO_3_‐based nanoantigens for O and pO, or for HOA and pOA, were determined using a bicinchoninic acid protein assay kit and a dsDNA quantification kit. The results indicated that the loading efficiency for the pO or pOA plasmid was approximately 90% in all cases. In contrast, the loading efficiency for HOA (ranging from 19% to 21%) was slightly lower than that for O (ranging from 22% to 24%, Figure 1K). In summary, these preliminary findings indicate that the O antigen modified with the E3 ligase‐recruiting ALAPYIP peptide more effectively enhanced the presentation of MHC I‐O_257‐264_ on DCs compared to the unmodified O antigen. Moreover, the successful preparation of HpOAC was achieved, and its morphological and crystalline characteristics were found to be conducive to promoting XPT in DCs. It should be noted that all subsequent comparisons among different nanoantigen formulations were performed using the same total nanoparticle concentration.

### 2.2 HpOAC Promotes DC Maturation and Migration Mediated by Ca^2+^

**FIGURE 2:**
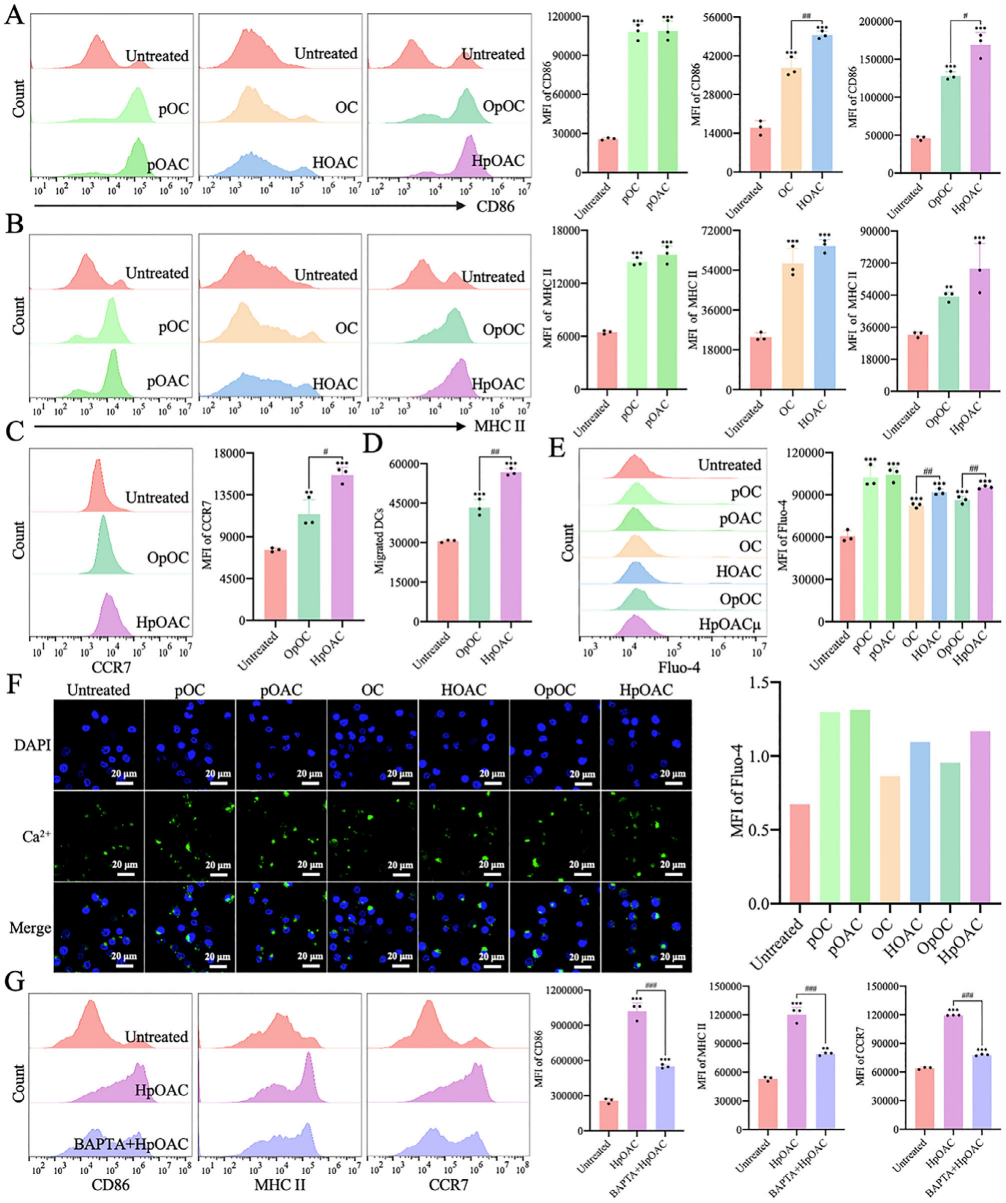
The adjuvant effects of HpOAC are mediated by CaCO_3_. (A, B) After treatment of DCs with pOC, pOAC, OC, HOAC, OpOC, and HpOAC for 24 h, the expression levels of CD86 and MHC II on their surface were measured by FCM, along with statistical results; (C) After treatment of DCs with OpOC and HpOAC for 24 h, the expression level of CCR7 on their surface was measured; (D) After 4 h, the number of DCs detected by FCM migrated from the Transwell upper chamber containing complete medium without CCL19 to the lower chamber, which was supplemented with complete medium containing 50 ng/mL CCL19, DCs were pretreated with OpOC and HpOAC for 24 h; (E, F) Cytosolic Ca^2+^ levels in DCs treated with different nanoantigens were detected by FCM and confocal laser scanning microscopy; (G) After pretreatment of DCs with the Ca^2+^ chelator BAPTA, the cells were stimulated with HpOAC for 24 h, and the expression levels of CD86, MHC II, and CCR7 on their surface were then measured by FCM. ^*^ *p* < 0.05, ^**^ *p* < 0.01, ^***^ *p* < 0.001 compared with untreated group by ANOVA. ^#^ *p* < 0.05, ^##^ *p* < 0.01, ^###^ *p* < 0.001 compared with both groups by unpaired *t*‐tests.

Based on our previous finding that DCs maintained high viability upon stimulation with 50 µg/mL of CaCO_3_‐based nanoantigens [18], we chose this concentration as the optimal dose for subsequent experiments. DCs were stimulated with pOC, pOAC, OC, HOAC, OpOC, or HpOAC for 24 h, before their expression levels of the costimulatory molecule CD86 and the antigen‐presenting molecule MHC II were assessed by FCM. The results showed that all nanoantigen formulations effectively promoted DC maturation. Notably, HOAC and HpOAC activated DCs more efficiently than OC and OpOC did, while the adjuvant effects of pOC and pOAC were comparable (Figure 2A,B). Subsequently, the expression level of C‐C motif chemokine receptor 7 (CCR7) on the surface of DCs and their migratory ability were examined after 24 h of stimulation with OpOC or HpOAC. The results demonstrated that both treatments enhanced CCR7 expression and migration of DCs, with HpOAC promoting migration to a significantly greater extent than that promoted by OpOC (Figure 2C,D). To investigate the mechanism underlying the differences in these adjuvant effects, we first measured the cytosolic Ca^2+^ levels in DCs following stimulation with various nanoantigens at the same dose using FCM with a calcium ion fluorescent probe. The results showed that the cytosolic Ca^2+^ levels in the pOC and pOAC groups were comparable and higher than those in the other groups. In contrast, the Ca^2+^ levels in the HOAC and HpOAC groups were higher than those in the OC and OpOC groups, respectively (Figure 2E). This pattern was consistent with the antigen loading efficiencies shown in Figure 1K: at the same tested dose (50 µg/mL), lower antigen loading corresponded to higher Ca^2+^ levels. In addition, we validated the FCM results using confocal laser scanning microscopy, which preliminarily indicated that the HpOAC‐mediated promotion of DC maturation and migration is Ca^2+^‐dependent (Figure 2F). Furthermore, we investigated the kinetics of Ca^2+^ release in DCs treated with HpOAC for 3, 6, 12, 18, and 24 h. The results showed that cytosolic Ca^2+^ levels began to increase after 6 h and peaked at 18 h, during which DC maturation, lysosomal acidification and membrane disruption occurred [21, 22], collectively exhibiting time‐dependent and pH‐responsive characteristics (Figure S6). Importantly, DCs were pretreated with BAPTA (Ca^2+^ chelator) prior to stimulation with HpOAC. The results showed that the surface expression levels of CD86, MHC II, and CCR7 were significantly reduced in the BAPTA‐pretreated group compared to the group treated with HpOAC alone, demonstrating that the adjuvant effect of HpOAC is mediated by the CaCO_3_ nanocarrier (Figure 2G).

### 2.3 DNA/PROTAC Characteristics Endow HpOAC With a Stronger Ability of XPT

**FIGURE 3:**
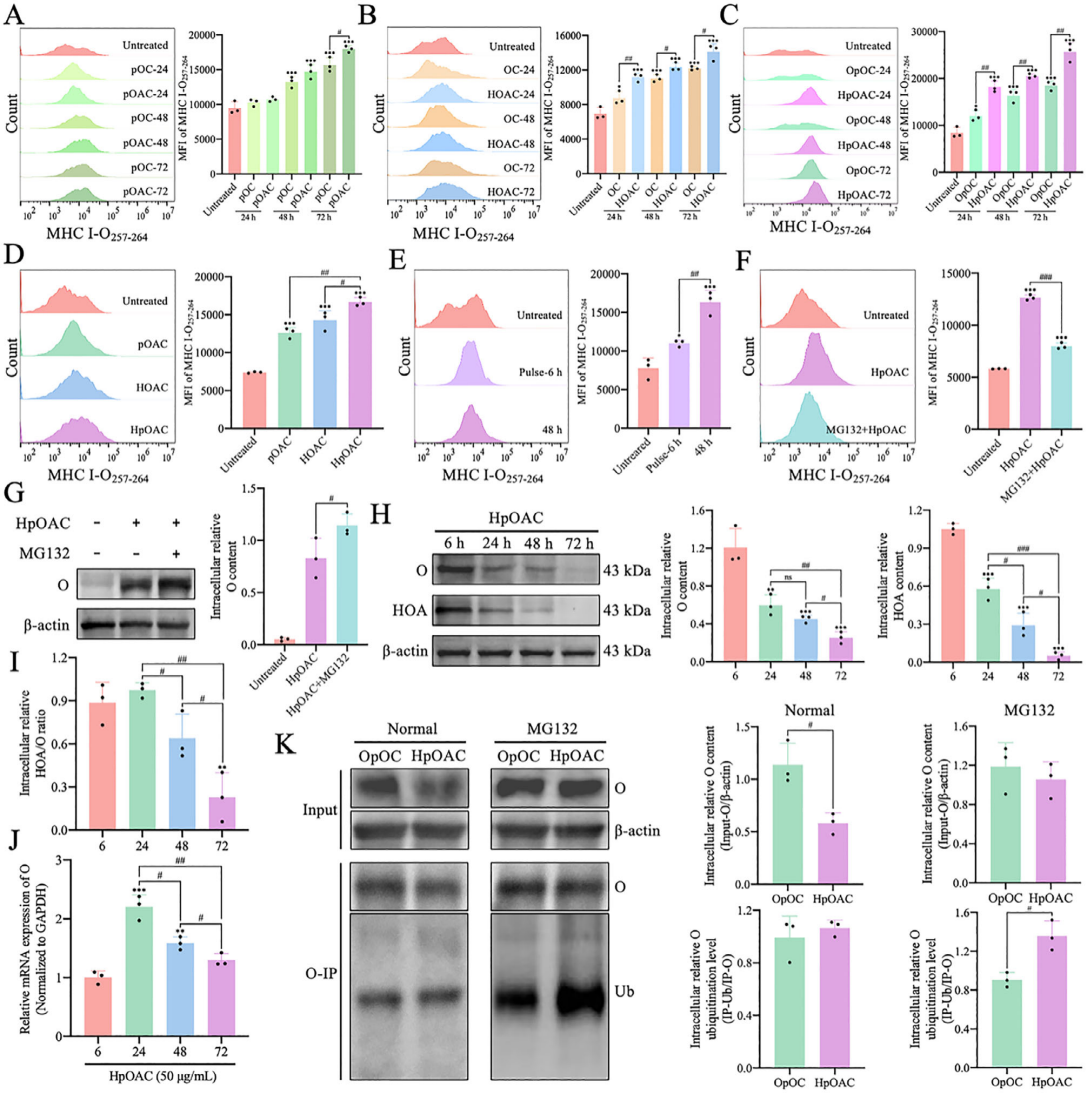
The mechanism by which HpOAC promotes XPT in DCs. (A) After stimulation of DCs with pOC and pOAC for 24, 48, and 72 h, respectively, the expression levels of MHC I‐OVA_257‐264_ on the DC surface were detected by FCM, along with statistical result; (B) The experimental procedure was consistent with that of A, with the exception that the nanoantigens used to stimulate DCs were replaced with OC and HOAC; (C) The experimental procedure was consistent with that of A and B, with the exception that the nanoantigens used to stimulate DCs were replaced with OpOC and HpOAC; (D) After stimulation of DCs with pOAC, HOAC, and HpOAC for 48 h, the expression level of MHC I‐OVA_257‐264_ on the DC surface was measured, along with statistical result; (E) After DCs were pulsed with HpOAC for 6 h or continuously stimulated for 48 h, the expression level of MHC I‐OVA_257‐264_ on the DC surface was measured, along with statistical result; (F) DCs were pretreated with the proteasome inhibitor MG132 for 3 h and then stimulated with HpOAC for 48 h, after which the XPT levels in DCs were detected by flow cytometry, along with statistical result; (G) After pretreatment of DCs with MG132 and subsequent stimulation with HpOAC, cytosolic O levels were detected through WB. Statistical data was performed by scanning the gray values of O and β‐actin bands using ImageJ, and the results were expressed as the ratio of O to β‐actin; (H) After stimulation of DCs with HpOAC for 6, 24, 48, and 72 h, the levels of total O and the prokaryotic recombinant protein HOA in the DC cytoplasm were detected by WB. The gray values of O and HOA were each normalized to that of β‐actin, and the resulting ratios were statistically analyzed; (I) The result was calculated by dividing the normalized gray value of HOA by that of O; (J) After stimulation of DCs with HpOAC for 6, 24, 48, and 72 h, the mRNA expression level of O was detected by quantitative reverse transcription polymerase chain reaction; (K) After pretreatment with MG132, DCs were stimulated with OpOC and HpOAC for 48 h, respectively. The ubiquitination levels of O were then detected by WB and co‐immunoprecipitation, using the group without MG132 pretreatment as a control. ^*^ *p* < 0.05, ^**^ *p* < 0.01, ^***^ *p* < 0.001 compared with untreated group by ANOVA. ^#^ *p* < 0.05, ^##^ *p* < 0.01, ^###^ *p* < 0.001 compared with both groups by unpaired *t*‐tests.

To evaluate the kinetics of XPT, DCs were stimulated with different nanoantigen formulations and assessed at 24, 48, and 72 h post‐treatment. pOC and pOAC induced no obvious XPT of DCs at 24 h, but both significantly enhanced their XPT at 48 h, with comparable efficacy. By 72 h, while both formulations continued to promote XPT, a significant difference between the pOC and pOAC groups occurred, indicating time‐dependent XPT enhancement by the DNA‐CaCO_3_ nanoantigen (Figure 3A). In parallel, OC and HOAC both significantly enhanced XPT at 24 h, with HOAC exerting a markedly stronger effect than OC. At later time points (48 and 72 h), both continued to promote XPT in a time‐dependent manner, although the difference between the two groups diminished relative to the 24 h time point (Figure 3B). Finally, OpOC and HpOAC both significantly enhanced XPT at all time points examined, with HpOAC consistently outperforming OpOC (Figure 3C). These results demonstrated that the PROTAC‐based strategy markedly potentiates antigen XPT by DCs. Additionally, we stimulated DCs with equivalent doses (50 µg/mL) of HOA, pOA, HOA+pOA, or HpOAC for 48 h and found that, compared with particulate HpOAC, soluble HOA and pOA were unable to promote antigen XPT in DCs (Figure S7). Subsequently, to validate the efficacy of different nanoantigen formulations—specifically DNA‐based (pOAC), protein‐based (HOAC), and protein + DNA‐based (HpOAC) nanoantigens—in promoting XPT, DCs were stimulated with each formulation for 48 h. The results demonstrated that the HpOAC group exhibited the strongest enhancement of XPT, which was significantly greater than that of the HOAC and pOAC groups, indicating that the protein + DNA‐integrated vaccine strategy markedly promotes XPT in DCs (Figure 3D). Furthermore, we investigated whether pulsing DCs with HpOAC could achieve XPT efficacy comparable to that of continuous stimulation. The results showed that a 6 h pulse with HpOAC resulted in significantly weaker XPT enhancement than that with continuous stimulation for 48 h (Figure 3E).

To elucidate the mechanism by which HpOAC promotes XPT in DCs, we first pretreated DCs with MG132 (a proteasome inhibitor), followed by stimulation with HpOAC for 48 h. The results showed that surface expression of MHC I‐O_257‐264_ was significantly reduced in the MG132‐pretreated group, indicating that HpOAC‐enhanced XPT is proteasome‐dependent (Figure 3F). Subsequently, using the same treatment regimen, we examined intracellular O levels in DCs by WB (Figure 3G). Corresponding to the FCM findings, O levels were markedly elevated in the MG132‐pretreated HpOAC group compared to the control group (treated with HpOAC alone). Under the same conditions, we compared the cytosolic O levels in DCs treated with either HpOAC or a combination of naked HOA protein and pOA plasmid transfected using Lipofectamine 3000. The results showed that the O level was significantly higher in the HpOAC group than in the HOA + Lipo‐pOA group, which can be attributed to the fact that particulate HpOAC is more readily phagocytosed by DCs than soluble HOA (Figure S8). Furthermore, DCs were stimulated with HpOAC for 6, 24, 48, and 72 h, after which the cytosolic levels of total O and the prokaryotically expressed recombinant HOA were assessed by WB. The results showed that HOA abundance was markedly reduced after 48 h, whereas total O bands remained detectable up to 72 h (Figure 3H). This observation was further supported by comparison of the normalized HOA and O levels, indicating that the presence of the DNA antigen (pOA plasmid) increases protein antigen abundance in the cytosol and prolongs the immunogenicity of DCs (Figure 3I). In order to directly confirm the functional role of pOA, the mRNA expression levels of O in DCs were detected at 6, 24, 48, and 72 h. The results showed that mRNA expression peaked at 24 h and then progressively decreased at 48 and 72 h, which corresponded to the WB findings, indicating that after 24 h, the mRNA was translated into protein, which then gradually diminished (Figure 3J). Importantly, we further assessed the ubiquitination level of O by Co‐IP after pretreating DCs with MG132 (the titration and specificity validation of the anti‐O antibody for IP are shown in Figure S9). In the absence of MG132, the O level in the Input fraction was significantly lower in the HpOAC group than in the OpOC group, whereas the ubiquitin (Ub) level in the IP fraction was comparable between the two groups, which can be attributed to the degradation of ubiquitinated O (Figure 3K). In contrast, upon MG132 pretreatment, the O level in the Input fraction was similar between the HpOAC and OpOC groups, but the Ub level in the IP fraction was markedly higher in the HpOAC group than in the OpOC group (Figure 3K). These results demonstrate that the presence of the E3 ligase‐recruiting ALAPYIP peptide promotes the ubiquitination of O.

### 2.4 HpOAC‐DCs Have Stronger Immunogenicity Compared With pOAC/HOAC‐DCs

**FIGURE 4:**
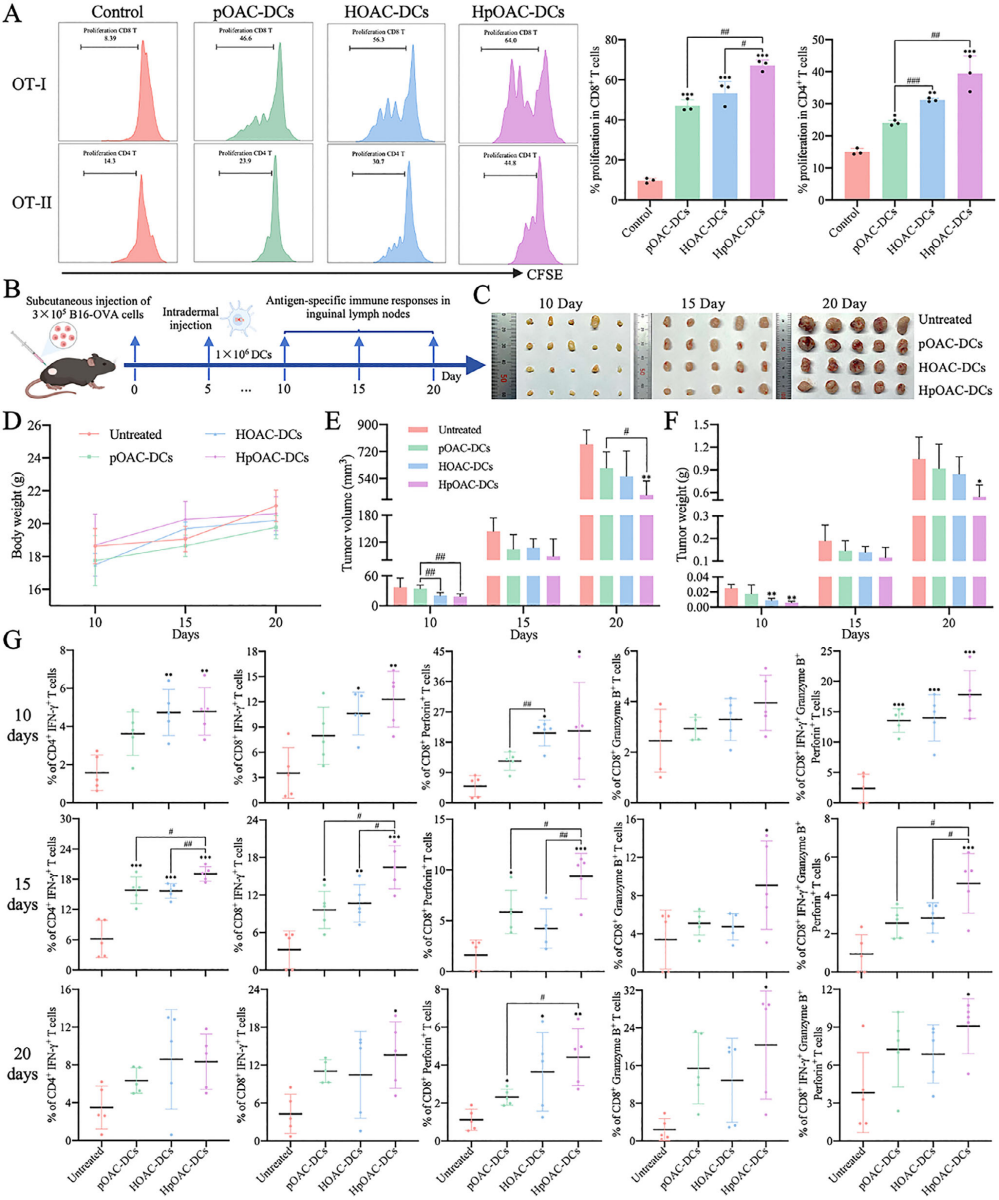
The DC vaccine integrating protein and DNA exhibits enhanced immunogenicity. (A) After stimulation of DCs with pOAC, HOAC, and HpOAC for 48 h, respectively, they were co‐cultured for 72 h with T cells sorted from the spleens of OT‐I or OT‐II mice. The proliferation of CD8^+^ or CD4^+^ T cells was then detected by FCM, and the data were presented as the percentage of CFSE^low^ cells; (B) Schematic diagram of the experimental procedure for the B16‐OVA tumor model in C57BL/6 mice, including tumor implantation and vaccination (*n* = 5); (C) Dissected tumors from mice on days 10, 15, and 20; (D) Body weight growth curves of tumor‐bearing mice; (E, F) Tumor volume and weight of tumor‐bearing mice; (G) Antigen‐specific Th1‐type and CTL immune responses in the inguinal lymph nodes of tumor‐bearing mice on days 10, 15, and 20. ^*^ *p* < 0.05, ^**^ *p* < 0.01, ^***^ *p* < 0.001 compared with untreated group by ANOVA. ^#^ *p* < 0.05, ^##^ *p* < 0.01, ^###^ *p* < 0.001 compared with both groups by unpaired *t*‐tests.

To evaluate the immunogenicity of DC vaccines prepared with different nanoantigen formulations, T cell proliferation assays were performed in vitro. The results showed that all DC vaccines made from various nanoantigen formulations promoted the proliferation of both CD8^+^ and CD4^+^ T cells (Figure 4A). Notably, the proliferation effect on CD8^+^ T cells (50%–70%) was more pronounced than that on CD4^+^ T cells (25%–40%) for all DCs stimulated by CaCO_3_‐based nanoantigens (Figure 4A). This is attributable to the properties of CaCO_3_ as a pH‐responsive delivery system, which responds to endosomal acidification during DC maturation, facilitating antigen escape from lysosomes into the cytoplasm for proteasomal degradation, leading to subsequent MHC I presentation and recognition by CD8^+^ T cells. Interestingly, in either of these T cell subsets, the HpOAC‐DCs exhibited significantly stronger proliferation‐promoting capacity than pOAC‐DCs or HOAC‐DCs, indicating that the DC vaccine prepared with the protein/DNA‐integrated nanoantigen possesses superior immunogenicity (Figure 4A).

We next examined the persistence of immunogenicity induced by HpOAC‐DCs in vivo. Tumor‐bearing mice were immunized once on day 5 after inoculation with B16‐OVA cells, and the draining inguinal lymph nodes were subsequently collected on days 10, 15, and 20 to assess antigen‐specific immune responses (Figure 4B). Notably, both tumor volume and weight were lower in the HpOAC‐DCs group than in the pOAC‐DCs and HOAC‐DCs groups, whereas no significant difference was observed between the pOAC‐DCs and HOAC‐DCs groups (Figure 4C–F). Furthermore, analysis of antigen‐specific immune responses in inguinal lymph nodes revealed that DC vaccines prepared with all three antigen formulations elicited stronger activation of Th1‐type responses and CTL activity on days 10–15 than on day 20. Among the groups, HpOAC‐DCs induced the most potent and durable immune responses overall (Figure 4G). Collectively, the above results conclusively demonstrated the superior anti‐tumor efficacy of DC vaccines prepared with the DNA/protein‐integrated antigen compared to those generated with single‐component antigens.

### 2.5 HpOAC‐DCs are More Capable of Inhibiting Tumor Growth Than OpOC‐DCs

**FIGURE 5:**
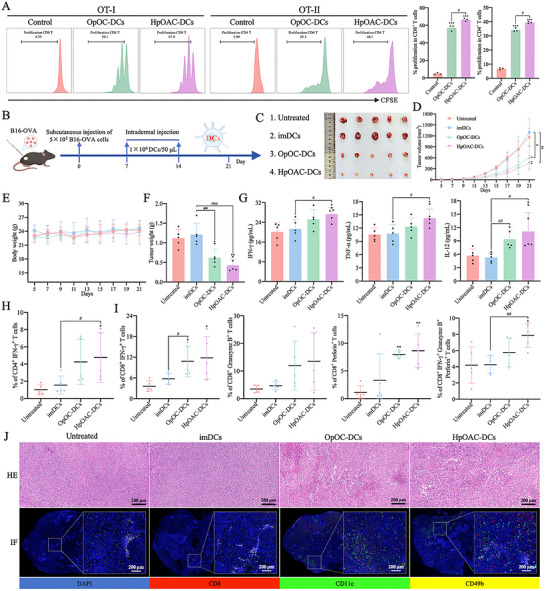
PROTAC enhances the anti‐tumor efficacy of HpOAC‐DCs. (A) After stimulation of DCs with OpOC, and HpOAC for 48 h, respectively, they were co‐cultured for 72 h with T cells sorted from the spleens of OT‐I or OT‐II mice. The proliferation of CD8^+^ or CD4^+^ T cells was then detected by FCM, and the data were presented as the percentage of CFSE^low^ cells; (B) Schematic diagram of the experimental procedure for the B16‐OVA tumor model in C57BL/6 mice, including tumor implantation and vaccination (*n* = 5); (C) Images of dissected tumors; (D‐F) Tumor volume, body weight, and tumor weight of tumor‐bearing mice; (G) The serum levels of the cytokines IFN‐γ, TNF‐α, and IL‐12 in tumor‐bearing mice were measured using ELISA kits; (H, I) Antigen‐specific immune responses in the inguinal lymph nodes of tumor‐bearing mice to O, with intracellular and surface staining of CD3, CD4, CD8, IFN‐γ, Granzyme B, and Perforin for FCM analysis; (J) HE pathological sections of the dissected tumor tissues and immunofluorescence images of T cells, DCs, and NK cells. ^*^ *p* < 0.05, ^**^ *p* < 0.01, ^***^ *p* < 0.001 compared with untreated group by ANOVA. ^#^ *p* < 0.05, ^##^ *p* < 0.01, ^###^ *p* < 0.001 compared with both groups by unpaired *t*‐tests.

Our previous study demonstrated that small‐molecule PROTAC‐conjugated antigens confer enhanced immunogenicity and anti‐tumor efficacy to DCs [18]. To investigate whether a peptide‐based PROTAC exerts similar effects, we compared the capacity of OpOC‐DCs and HpOAC‐DCs to promote T cell proliferation. The results showed that both HpOAC‐DCs and OpOC‐DCs significantly enhanced the proliferation of CD8^+^ and CD4^+^ T cells, with the HpOAC‐DCs group exhibiting a significantly higher proliferation rate than the OpOC‐DCs group (Figure 5A). These findings indicate that fusion of the ALAPYIP peptide with the antigen similarly enhances the immunogenicity of DCs. Moreover, consistent with the targeted enhancement of XPT, both formulations exhibited a stronger activating effect on CD8^+^ T cells than on CD4^+^ T cells (Figure 5A).

To demonstrate the superior anti‐tumor efficacy of PROTAC‐enhanced HpOAC‐DCs over OpOC‐DCs, tumor‐bearing mice were immunized twice with HpOAC‐DCs or OpOC‐DCs on days 7 and 14, using imDCs as a control. All mice were euthanized on day 21 for subsequent physiological and immunological analyses (Figure 5B). The results revealed that organ indices remained stable across all groups (Figure S10). The body weight of mice showed no significant fluctuations, and tumor volume and weight were significantly lower in the HpOAC‐DCs and OpOC‐DCs groups than in the untreated and imDCs groups, with the HpOAC‐DCs group exhibiting slightly lower values compared to the OpOC‐DCs group (Figure 5C–F). Subsequently, the serum levels of cytokines including IFN‐γ, TNF‐α, and IL‐12 in tumor‐bearing mice were measured by ELISA. The results revealed that the HpOAC‐DCs and OpOC‐DCs groups exhibited significantly higher cytokine levels compared to the untreated and imDCs groups, with the HpOAC‐DCs group showing slightly elevated levels relative to the OpOC‐DCs group (Figure 5G). Furthermore, immunophenotyping of immune cells in the spleen revealed that the numbers of activated DCs (CD11c^+^ CD86^+^), macrophages (CD11b^+^ CD86^+^), central memory T cells (CD4^+^ CD44^+^ CD62L^+^ and CD8^+^ CD44^+^ CD62L^+^), effector memory T cells (CD4^+^ CD44^+^ CD62L^−^ and CD8^+^ CD44^+^ CD62L^−^), B cells (CD3^−^ CD19^+^), and NK cells (CD3^−^ CD19^−^ CD49b^+^) were higher in the HpOAC‐DCs group than in the OpOC‐DCs group (Figure S11). In addition, the number of regulatory T cells (Tregs, CD3^+^ CD4^+^ Foxp3^+^) in the HpOAC‐DCs group showed a decreasing trend compared with the other groups (Figure S11). Collectively, these results indicate that the immune system of tumor‑bearing mice in the HpOAC‑DCs group was activated, and the increased numbers of central and effector memory T cells suggest that HpOAC‑DCs can more effectively establish immunological memory in tumor‑bearing mice. Importantly, antigen‐specific immune responses in the inguinal lymph nodes of tumor‐bearing mice were assessed, yielding results consistent with the findings described above. Both the HpOAC‐DCs and OpOC‐DCs groups exhibited significantly stronger immune responses than the untreated and imDCs groups, with the HpOAC‐DCs group showing a slight advantage over the OpOC‐DCs group (Figure 5H,I). Finally, histological analysis of tumor tissues by H&E staining and immunofluorescence revealed more pronounced tissue damage and enhanced infiltration of DCs, T cells, and NK cells in the HpOAC‐DCs and OpOC‐DCs groups compared to controls (Figure 5J). Overall, the HpOAC‐DCs group exhibited slightly greater anti‐tumor immune cell infiltration and tissue damage than the OpOC‐DCs group (Figure 5J). In summary, HpOAC‐DCs demonstrated slightly greater anti‐tumor efficacy compared to OpOC‐DCs. Although this enhancement was modest, it indicates that the peptide‐based PROTAC strategy can further enhance antigen XPT in DCs.

### 2.6 HpOAC‐DCs Vaccine Has Better Anti‐Tumor Efficacy Than That of HpOAC‐Ag Vaccine

**FIGURE 6:**
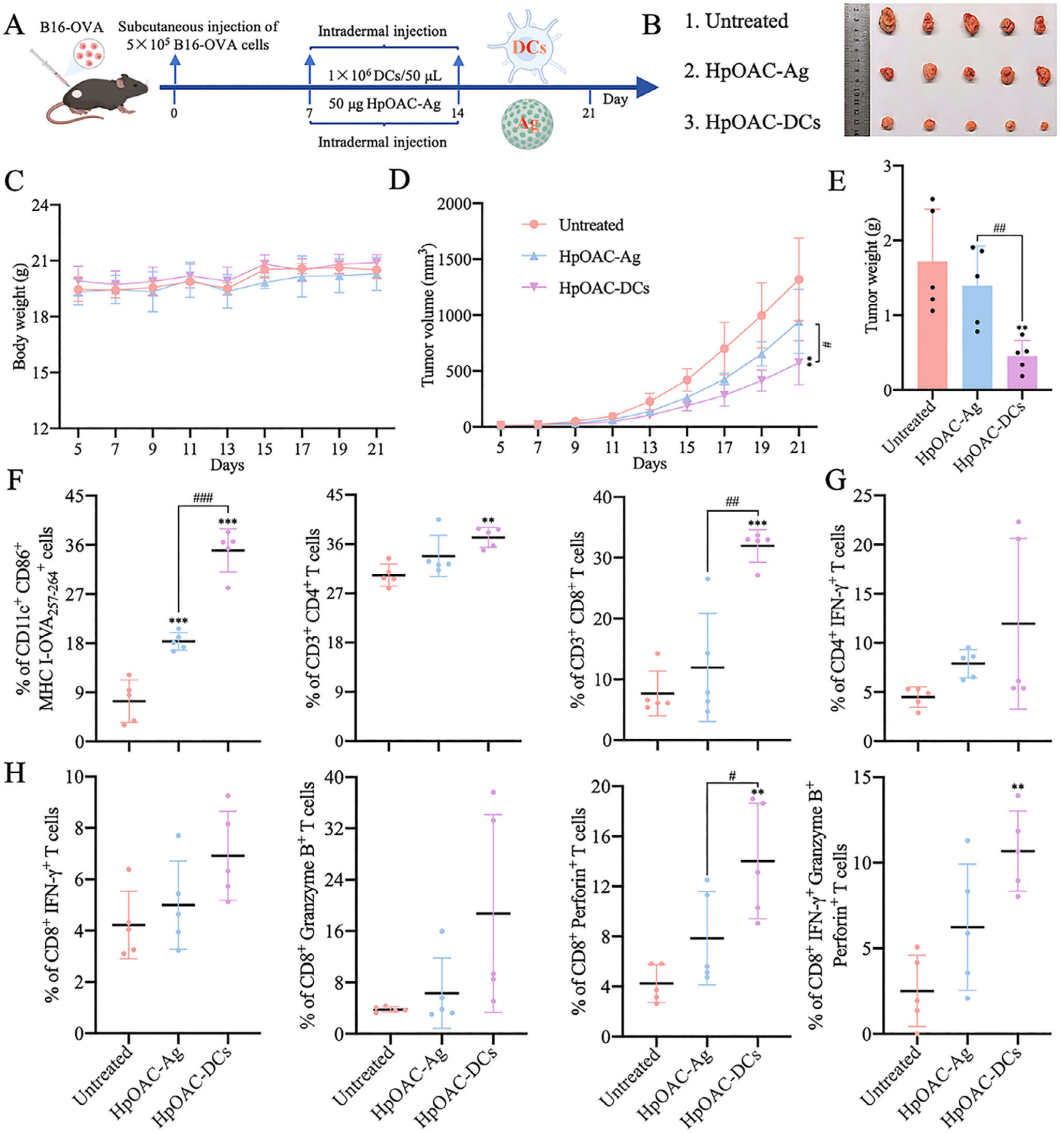
Anti‐tumor efficacy of HpOAC‐Ag and HpOAC‐DCs. (A) The vaccination schedule of HpOAC‐DCs and HpOAC‐Ag on the experimental procedure of B16‐OVA tumor mouse model (*n* = 5); (B) Images of dissected tumors; (C‐E) Body weight, tumor volume, and tumor weight of tumor‐bearing mice; (F) The proportions of activated DCs, as well as CD4^+^ and CD8^+^ T cells, in the inguinal lymph nodes of tumor‐bearing mice; (G, H) Antigen‐specific immune responses in the inguinal lymph nodes of tumor‐bearing mice to OVA. ^*^ *p* < 0.05, ^**^ *p* < 0.01, ^***^ *p* < 0.001 compared with untreated group by ANOVA. ^#^ *p* < 0.05, ^##^ *p* < 0.01, ^###^ *p* < 0.001 compared with both groups by unpaired *t*‐tests.

To verify that the anti‐tumor efficacy of HpOAC‐DCs is primarily mediated by the DC vaccine rather than by the HpOAC as antigen, we evaluated the anti‐tumor effect of HpOAC (HpOAC‐Ag) administered as an in situ vaccine in a mouse B16‐OVA tumor model, using HpOAC‐DCs as a control (Figure 6A). The results showed that organ indices and body weight remained stable across groups, whereas tumor volume and weight were significantly lower in the HpOAC‐DC group than in the HpOAC‐Ag group (Figure S12, Figure 6B–E). Subsequently, the proportions of activated DCs, CD4^+^ T cells, and CD8^+^ T cells in the inguinal lymph nodes of mice were assessed. The results showed that the HpOAC‐DC group exhibited significantly higher levels of activated DCs and CD8^+^ T cells compared to the HpOAC‐Ag group (Figure 6F). Notably, the gating strategy for activated DCs in Figure 6F specifically identified CD11c^+^ CD86^+^ MHC I‐O_257‐264_^+^ cells. While HpOAC‐Ag administration significantly enhanced XPT by endogenous DCs compared to the untreated group, this effect was markedly weaker than that achieved by HpOAC‐DCs. Importantly, antigen‐specific immune responses in the inguinal lymph nodes revealed that both Th1‐type and CTL responses were stronger in the HpOAC‐DCs group than in the HpOAC‐Ag group (Figure 6G,H). This can be attributed to the fact that HpOAC‐DCs possess costimulatory capacity—which HpOAC‐Ag lacks—as well as enhanced active migratory property.

## 3 Discussion

Our previous experimental results in anti‐tumor DC vaccine design demonstrated that combining PROTAC technology with the CaCO_3_ nano‐delivery system successfully diverted larger amounts of foreign protein antigen to the cytosolic degradation pathway for MHC I presentation [18], and that incorporating recombinant eukaryotic and prokaryotic dual‐expression systems augmented both MHC I and MHC II presentation pathways [19]. Building upon these findings, we herein advanced the DC vaccine strategy by integrating both protein and DNA antigen formats into a unified PROTAC design. This design prokaryotically expresses the HOA fusion protein and eukaryotically expresses the pOA plasmid, which are then co‐delivered to DCs within CaCO_3_ nanoparticles as persistent antigen sources. The key advantage of this novel design is that it provides both immediate antigen supply (via the prokaryotically expressed protein) and sustained antigen supply (via the eukaryotically expressed plasmid). These antigen sources undergo degradation through both the MHC II pathway (for a portion of the prokaryotically expressed protein) and the MHC I pathway (for both the prokaryotically expressed protein and the eukaryotically expressed plasmid), thereby activating CD4^+^ and CD8^+^ T cells, respectively. This synergistic activation of tumor‐specific T cells, especially Th1 and CTL responses in vivo by this super DC vaccine, ultimately led to superior anti‐tumor efficacy compared to any other currently available vaccines, whether they are PROTAC‐based, protein or DNA antigen vaccines, or normal DC vaccines. This integrated protein/DNA DC vaccine design further potentiates the immunogenicity and durability of DC‐mediated anti‐tumor responses, offering a novel paradigm for DC‐based cancer immunotherapy.

The rationale for incorporating the ALAPYIP peptide into the PROTAC design is based on its well‐established capacity to recruit the VHL [23, 24]. Specifically, ALAPYIP is derived from the minimal amino acid sequence in hypoxia‐inducible factor 1α (HIF‐1α) that is capable of binding to VHL [25, 26, 27]. In 2009, Tang, YQ. et al. used the androgen receptor (AR) ligand dihydrotestosterone (DHT) together with ALAPYIP to construct DHT‐PROTAC, which significantly degraded AR in lymph node carcinoma of the prostate (LNCaP) cells. Competition experiments showed that free ALAPYIP peptide dose‐dependently blocked DHT‐PROTAC‐induced AR degradation, confirming that ALAPYIP competes with the ALAPYIP moiety in DHT‐PROTAC for binding to VHL [28]. Importantly, Si, LL. et al. utilized the ALAPYIP peptide as a VHL‐recruiting motif and fused it with viral proteins to construct attenuated vaccines. They found that proteasome‐dependent degradation of the fusion protein was completely abolished in VHL‐knockout Madin‐Darby canine kidney (MDCK) cells and human embryonic kidney 293T (HEK293T) cells compared with normal MDCK and HEK293T cells, indicating that the degradation of the fused viral protein depends on the recruitment of VHL by ALAPYIP, thereby providing direct functional evidence for the ALAPYIP‐VHL interaction [23, 24].

The selection of CaCO_3_ as the nanocarrier is guided by its dual functionality in facilitating antigen delivery and serving as an immunostimulatory adjuvant [21, 29, 30]. As documented in previous studies and our own work, the process of CaCO_3_‐mediated antigen delivery to DCs is accompanied by a series of events, including DC maturation, reactive oxygen species (ROS) elevation, endosomal acidification, CaCO_3_ decomposition, CO_2_ production, Ca^2+^ release, and subsequent lysosomal escape of antigens [18, 21, 22]. Our data suggest that Ca^2+^ release from HpOAC contributes directly to DC maturation and migration (Figure 2E–G), likely through Ca^2+^‐dependent signaling pathways such as nuclear factor kappa‐light‐chain‐enhancer of activated B cells (NF‐κB), a core pathway for innate immune activation or nuclear factor of activated T cells (NFAT), which are known to regulate costimulatory and chemokine receptor expression [29, 31]. However, a more careful interpretation of the role of CaCO_3_ is warranted. While BAPTA‐mediated Ca^2+^ chelation significantly reduces CD86, MHC II, and CCR7 expression (Figure 2G), it does not completely abrogate DC activation, suggesting that additional mechanisms—such as pH buffering, endosomal membrane disruption, and ROS elevation—may also contribute to the adjuvant effect. Nevertheless, our loss‐of‐function experiments with BAPTA demonstrate that Ca^2+^ plays a non‐redundant role in HpOAC‐induced DC maturation and migration. Collectively, CaCO_3_ acts through a multimodal mechanism, and Ca^2+^‐dependent signaling constitutes a key, but not exclusive, driver of the enhanced immunogenicity observed in the HpOAC platform. Future studies employing more selective inhibitors or genetic models are warranted to dissect the relative contributions of each pathway.

Mechanistically, the superior capacity of HpOAC to promote XPT was attributed to the synergistic interplay between its protein and DNA components, as well as the enhancement conferred by PROTAC. The preformed recombinant HOA protein provided an “initial burst” for XPT, whereas the pOA plasmid served as a “reservoir” for sustained antigen supply. As a result, HpOAC‐DCs not only significantly enhanced the proliferation of both CD4^+^ and CD8^+^ T cells in vitro, but also potently activated Th1‐type and CTL immune responses in vivo, thereby enhancing the anti‐tumor potency of the Th1‐CTL positive feedback loop. This functional superiority was similarly attributed to the integration of the two distinct antigen formats. Furthermore, Co‐IP experiments revealed that the presence of ALAPYIP significantly enhanced the ubiquitination of O, providing direct biochemical evidence for the role of PROTAC in empowering antigen XPT. Importantly, the therapeutic advantage of HpOAC‐DCs over OpOC‐DCs, though statistically significant, was modest in magnitude; nevertheless, it supports the added value of incorporating the VHL‐recruiting ALAPYIP peptide into the antigen design framework. Moreover, we chose plasmid DNA (pOA) rather than mRNA as the sustained antigen source because: (i) the immediate burst of antigen expression is already provided by the preformed HOA protein; (ii) plasmid DNA is more stable, has a longer half‐life, and is easier to formulate with CaCO_3_ (loading efficiency ∼90%); and (iii) we observed persistent OVA expression up to 72 h post‐stimulation (Figure 3H–J), supporting sustained immunogenicity.

While this study demonstrated the feasibility and efficacy of a PROTAC‐functionalized protein/DNA‐integrated DC vaccine, several limitations warrant consideration for improvement. First, although the use of O as a model antigen is experimentally convenient, it does not fully recapitulate the physicochemical properties, processing kinetics, or immunological context of true tumor neoantigens within the complex immune network in vivo, and therefore our conclusions regarding anti‐tumor efficacy are primarily established in this model antigen system; extrapolation to patient‐derived neoantigens requires further validation. Fortunately, the modularity of the PROTAC platform allows for facile replacement of O with other antigens, enabling rapid customization of vaccines for different tumor types or even infectious diseases. Second, although our splenic data indicate systemic immune activation in tumor‑bearing mice—as evidenced by increased numbers of effector memory T cells and NK cells—future quantitative analyses of the tumor‑infiltrating CD8^+^/Treg cell ratio, M1/M2 macrophage polarization, and Granzyme B^+^/IFN‑γ^+^ cells in authentic tumor neoantigen models will provide more definitive evidence for antitumor efficacy. Third, the potential off‐target effects or autoimmune risks resulting from enhanced antigen XPT require long‐term systematic evaluation. We acknowledge that comprehensive safety assessments, including serum biochemistry, histopathology of major organs, and inflammatory cytokine profiling, were not performed in this exploratory study—a limitation that becomes particularly relevant given that enhanced XPT may potentiate off‑target T cell responses or autoimmunity. Therefore, comprehensive evaluation of in vivo inflammatory cytokine profiles, serum biochemistry, and organ damage status under authentic tumor neoantigen models represents our next urgent priority.

In summary, the present study established a mechanistically next‐generation DC vaccine platform that integrates PROTAC‐functionalized protein and DNA antigens within a CaCO_3_ nano‐delivery system, although its broad potential awaits further verification using tumor neoantigen models. By harnessing the complementary advantages of protein antigens for immediate presentation and plasmid antigens for sustained presentation, HpOAC‐DCs elicit robust and durable anti‐tumor immunity. Mechanistic insights into Ca^2+^‐mediated adjuvanticity and antigen degradation pathways for both MHC I and II presentation to activate a broad spectrum of antigen‐specific T cells provide a scientific foundation for further optimizing the anti‐tumor efficacy of DC vaccines. As the field of cancer immunotherapy continues to evolve, strategies that enhance the intrinsic capacity of DCs to process and present antigens will remain at the forefront of vaccine development. Our HpOAC platform represents a significant step toward realizing the full potential of DC‐based immunotherapies and warrants continued investigation in preclinical and, ultimately, clinical settings.

## 4 Materials and Methods

### 4.1 Plasmid Construction, Protein Expression and Purification

Gene synthesis was employed to obtain the His‐Ovalbumin‐ALAPYIP (HOA) DNA sequence (Bio‐Transduction Lab, *Wuhan*, *China*), and a prokaryotic expression plasmid for HOA was constructed using pET32a as the vector. The HOA‐pET32a plasmid was transformed into *Escherichia coli* (*E. coli*) DH5α competent cells for amplification. Following confirmation by DNA sequencing, the verified plasmid was subsequently transformed into *E. coli* BL21 for protein expression. The structure of HOA was modeled using AlphaFold. Subsequently, the conditions for HOA expression were optimized, including temperature, isopropyl β‐D‐1‐thiogalactopyranoside (IPTG) concentration, and induction time. Under optimized conditions, the culture was scaled up, and the BL21 cells were harvested and disrupted by ultrasonication. Finally, the HOA protein was purified using immobilized metal ion affinity chromatography (IMAC) via its His tag.

The DNA sequences encoding O and OA were obtained from the HOA‐pET32a plasmid by polymerase chain reaction (PCR) and subsequently inserted into the pcDNA3.1 eukaryotic expression vector using double digestion. The resulting plasmids, designated O‐pcDNA3.1 (pO) and OA‐pcDNA3.1 (pOA), were verified by nucleic acid gel electrophoresis and DNA sequencing. For the extraction of pO and pOA plasmids, we used an endotoxin‑free plasmid maxi‑prep kit (Tiangen, *China*); after purifying the HOA protein, we used a protein endotoxin removal kit (Beyotime, *China*) to eliminate endotoxins from the protein samples, ensuring that the endotoxin content in all antigens was below 0.1 EU/mg, to facilitate the subsequent preparation of nano‑antigens.

### 4.2 Cell Culture

The induction and culture of DCs were performed as described previously [19]. Briefly, the femurs and tibias were harvested from C57BL/6 mice, sterilized, and the bone marrow cells were subsequently flushed out with Roswell Park Memorial Institute (RPMI) 1640 medium. After washing, the bone marrow cells were cultured in RPMI‐1640 complete culture medium supplemented with 10% heat‐inactivated fetal bovine serum, 100 U/mL penicillin, 100 µg/mL streptomycin, and 20 ng/mL granulocyte/macrophage colony‐stimulating factor (GM‐CSF). On day 7, the cells were collected, and the differentiation phenotype of DCs was characterized by flow cytometry (FCM) after staining with antibodies against CD11c and MHC II for subsequent experiments. Lymphocytes were harvested from the spleen or inguinal lymph nodes of C57BL/6 mice and cultured in RPMI‐1640 complete medium without GM‐CSF. B16‐OVA cells were also cultured in RPMI‐1640 complete medium without GM‐CSF. All cells were cultured in a humidified atmosphere at 37°C with 5% CO_2_.

### 4.3 Transfection

DCs were seeded into 24‐well plates at a density of 1 × 10^6^ cells per well in 500 µL of complete culture medium and maintained for subsequent experiments. Lipofectamine 3000 transfection reagent (ThermoFisher, *USA*) and plasmid DNA (pO and pOA) were diluted separately in Opti‐MEM I reduced serum medium using 1.5 mL microcentrifuge tubes. P3000 enhancer reagent was then added to the diluted plasmid DNA, and the two solutions were combined and incubated at room temperature for 15 min to facilitate the formation of DNA‐liposome complexes. The complexes were added to the 24‐well plate containing DCs and incubated for 8 h. Subsequently, an additional 1 mL of complete culture medium was added, and the cells were further cultured for 72 h. Finally, the DCs were harvested, washed with PBS, and stained with an anti‐SIINFEKL antibody. The intracellular O expression and the surface levels of MHC I‐O_257‐264_ on DCs were then assessed by Western blotting (WB) and FCM, respectively.

### 4.4 The Preparation Procedure of HpOAC

All chemicals and reagents were purchased from commercial sources (Macklin, *Shanghai*, *China*) and were used as received without further purification. The preparation of OC/HOAC was mainly performed as previously described by Wang, S. et al. [22]. Briefly, O/HOA (1 wt.%) aqueous solution was mixed with CaCl_2_ (0.5 m) solution thoroughly. Then, Na_2_CO_3_ (0.5 m) solution was added dropwise in a stepwise manner, followed by vigorous stirring for 10 min. Finally, the product was collected by centrifugation.

The preparation of pOC and pOAC was performed following a classic Ca^2+^ transfection method [32, 33]. Briefly, plasmid was thoroughly mixed with CaCl_2_ solution (0.5 m). Subsequently, Na_2_CO_3_ solution (0.5 m) was slowly added dropwise to the Ca^2+^‐DNA mixture. After stirring for 10 min, the resulting product was collected by centrifugation.

The preparation of OpOC and HpOAC was carried out as follows. Briefly, pO or pOA was first thoroughly mixed with CaCl_2_ solution (0.5 m). Subsequently, O or HOA (1 wt.%) aqueous solution was added, and the mixture was stirred for 5 min. Finally, an equal volume of Na_2_CO_3_ (0.5 m) solution (relative to the CaCl_2_ solution) was slowly added dropwise. After vigorous stirring for 10 min, the resulting product was collected by centrifugation. All chemical operations were carried out in a sterile fume cupboard.

### 4.5 Physicochemical Characterization

The morphology of HpOAC was characterized using a high‐power field emission scanning electron microscope (SEM, SU8010, HITACHI, *Japan*). The crystal structure of HpOAC was characterized using an x‐ray diffractometer (XRD, D8, BRUKER, *Germany*). The particle size and zeta potential of HpOAC were measured using a nanoparticle size and Zeta potential analyzer (DLS, Zetasizer Nano ZS90, Malvern, *Britain*). The protein or plasmid loading rates were determined using the BCA Protein Quantification Kit (Yeasen, *China*) and the dsDNA HS Assay kit (Yeasen, *China*) through a multi‐functional microplate reader (SpectraMax iD5, Molecular Devices, *USA*). The loading rate was calculated using the following formula: (Total amount of protein or plasmid added – Amount of free protein or plasmid in the supernatant) / Total amount of protein or plasmid added × 100%.

### 4.6 Animals and Ethics

Female C57BL/6 and BALB/c mice aged 6–8 weeks were purchased from the Animal Laboratory Center of Xinjiang Medical University (*Urumqi, China*). All mice were maintained at the Laboratory Animal Center of Xinjiang University, with a controlled temperature and a standardized light–dark cycle. OT‐I and OT‐II transgenic mice were sourced from The Jackson Laboratory (*USA*) and housed under specific pathogen‐free (SPF) conditions at Xinjiang University. All animal experiments were approved by the Committee on the Ethics of Animal Experiments of Xinjiang Key Laboratory of Biological Resources and Genetic Engineering (XJUAE‐2025‐014), and conducted in accordance with the guidelines of the Animal Care and Use Committee of the College of Life Science and Technology, Xinjiang University.

### 4.7 Confocal Laser Scanning Microscopy

DCs (5 × 10^5^ cells) were seeded onto glass‐bottom confocal dishes and stimulated with pOC, pOAC, OC, HOAC, OpOC, or HpOAC for 24 h. After stimulation, unbound nanoantigens were removed by washing, and intracellular Ca^2+^ was labeled with Fluo‐4 AM (Beyotime, *China*). The cells were then visualized using a confocal laser scanning microscope (A1RHD25, Nikon, *Japan*). Fluorescence intensity was quantified using Image J software.

### 4.8 Flow Cytometry

All flow cytometry (FCM) was performed using a CytoFLEX (V4‐B4‐R3, BECKMAN COULTER, *USA*). FCM antibodies such as CD3 (17A2, APC‐A700), CD4 (GK1.5, PerCP), CD8 (53‐6.7, PE‐Cyanine7), CD19 (1D3, APC), CD49b (DX5, FITC), CD44 (IM7, PE), CD62L (DREG56, Violet610), CD11c (N418, FITC), CD11b (M1/70, PE), Gr‐1 (RB6‐8C5, PE‐Cyanine7), MHC II (M5/114, PE‐Cyanine7), CD40 (FGK4.5/FGK45, PerCP), CD86 (GL‐1, PE) were purchased from Elabscience (*Wuhan, China*). Foxp3 (FJK‐16s, FITC), CD25 (CD25‐4E3, PE), MHC I‐O_257‐264_ complex antibody (25‐D1.16, APC), IFN‐γ (4S.B3, APC), Granzyme B (NGZB, PE‐Cyanine7), Perforin (eBioOMAK‐D, FITC), and CCR7 (3D12, PE) antibodies were purchased from ThermoFisher. All flow cytometry gating strategies are shown in Figure S13.

### 4.9 DC Migration

After stimulation with HpOAC for 24 h, the surface expression of CCR7 on DCs was assessed by FCM. DC migration was assessed using a Transwell cell culture plate (CORNING, *USA*). This Transwell plate consists of an upper chamber and a lower chamber separated by a selectively permeable membrane. Cells in the upper chamber are attracted by chemokines in the lower chamber and migrate across the permeable membrane into the lower chamber. HpOAC‐treated DCs (5 × 10^5^/200 µL) were added to the upper chamber of a Transwell plate, and 500 µL of complete medium supplemented with 50 ng of C‐C motif chemokine ligand 19 (CCL19) was added to the lower chamber. Finally, cells that had migrated into the lower chamber of the Transwell plate were collected into 1.5 mL centrifuge tubes and counted.

### 4.10 XPT of Antigens by DCs

The expression of MHC I‐O_257‐264_ complexes on DCs treated with pOC, pOAC, OC, HOAC, OpOC, and HpOAC for 24, 48, and 72 h was detected by FCM. The expression of MHC I‐O_257‐264_ on DCs was assessed after pulsing with HpOAC for 6 h or continuous stimulation for 48 h. After pretreating DCs with 200 nm MG132 (a proteasome inhibitor that can effectively block the proteolytic activity of the 26S proteasome) for 3 h, the XPT of HpOAC in DCs was detected. The above experiments were performed in 24‐well plates, with cells seeded at a density of 1 × 10^6^ cells per well and treated with nanoantigens at a concentration of 50 µg/mL.

### 4.11 Western Blot

DCs were placed into 60 mm cell culture dish at a density of 1 × 10^7^/dish. After pretreating DCs with MG132 (a proteasome inhibitor), the DCs were stimulated with HpOAC for 48 h. Subsequently, DCs were collected, washed three times with PBS, and then lysed on ice with RIPA lysis buffer containing protease inhibitor and phosphatase inhibitor (SANGON, *Shanghai, China*) for 30 min. Finally, the cell lysates were centrifuged at 12 000 rpm for 20 min, and the supernatants were collected to obtain cytoplasmic proteins. Furthermore, DCs were harvested at 6, 24, 48, and 72 h post‐stimulation with HpOAC, and the intracellular levels of total O and HOA were assessed by WB. Antibodies against O and β‐actin, as well as goat anti‐rabbit IgG‐HRP, were purchased from Elabscience.

### 4.12 Quantitative Reverse Transcription Polymerase Chain Reaction (qRT‐PCR)

DCs were placed into 60 mm cell culture dishes at a density of 1 × 10^7^/dish and then stimulated with HpOAC for 6, 24, 48, and 72 h. After stimulation, the DCs were harvested, washed three times with PBS, and total RNA was extracted using an RNA extraction kit (FOREGENE, *China*). Finally, the relative mRNA levels of O at different time points were quantified by qRT‐PCR using a commercial kit (TaKaRa, *Japan*). All experiments were performed according to the manufacturer's instructions.

### 4.13 Ubiquitination Detection of O

The ubiquitination level of O in DCs was assessed by co‐immunoprecipitation (Co‐IP). DCs were pretreated with MG132 for 3 h to inhibit proteasomal degradation, thereby enriching ubiquitinated O for subsequent detection. DCs treated without MG132 or with OpOC were used as controls. After lysing DCs and collecting cytoplasmic proteins under non‐denaturing conditions, an excess of O‐specific IP antibody (200‐4633‐0100, ThermoFisher, *USA*) was added to the cell lysates. The mixture was incubated at 4°C for 8 h under gentle agitation to allow formation of the antibody‐O complex. Subsequently, Protein A/G Magnetic Beads (ThermoFisher) were added, and the mixture was incubated for an additional 2 h at room temperature to immunoprecipitate the antigen‐antibody complex. Finally, excess IP antibody was removed by washing, and the immunoprecipitated samples were then subjected to Western blotting using an anti‐ubiquitin antibody.

### 4.14 Proliferation of T Cell

After stimulation with HpOAC for 48 h, the DCs were collected. The spleen tissues from OT‐I and OT‐II mice were harvested and processed into single‐cell suspensions. T cells were then isolated using anti‐CD3 magnetic beads (Miltenyi Biotec, *Germany*) and subsequently labeled with carboxyfluorescein succinimidyl ester (CFSE, ThermoFisher) for proliferation analysis. Finally, DCs and T cells were co‐cultured at a 1:5 ratio, and T‐cell proliferation was assessed by FCM after 72 h.

### 4.15 Preparation of HpOAC‐DC Vaccine

The DC‐based vaccine was prepared as previously described [18]. Immature DCs (imDCs) without any treatment served as the negative group. DCs treated with pOC (pOC‐DCs), HOAC (HOAC‐DCs), and OpOC (OpOC‐DCs) were used as control groups, while DCs stimulated with 50 µg/mL HpOAC (HpOAC‐DCs) served as the experimental vaccine group. All DCs were stimulated with the indicated nanoantigens for 48 h. Direct intradermal injection of 50 µg naked HpOAC was used as an in situ vaccine (HpOAC‐Ag).

### 4.16 Animal Model

C57BL/6 mice were subcutaneously inoculated in the right flank with 3 × 10^5^ B16‐OVA cells. Five days later, the mice received a single intradermal injection of pOC‐DCs, HOAC‐DCs, or HpOAC‐DCs vaccine (1 × 10^6^ DCs/50 µL). On days 10, 15, and 20 post‐inoculation, body weight, tumor volume, and tumor weight were measured, and antigen‐specific immune responses in the inguinal lymph nodes were assessed.

5 × 10^5^ B16‐OVA cells were subcutaneously injected into the right flank of C57BL/6 mice. 7 days post‐inoculation, the tumor‐bearing mice were randomly assigned to different groups and treated with DC‐based vaccines or an in situ vaccine, including imDCs, OpOC‐DCs, HpOAC‐DCs, or HpOAC‐Ag. The vaccine was administered intradermally twice, with a one‐week interval between doses. On day 21, the mice were sacrificed and dissected to evaluate changes in pathological or immunological parameters. To assess the antigen‐specific immune response, inguinal lymph nodes were harvested from the tumor‐bearing side of mice treated with DC vaccines. The lymph nodes were then processed into a single‐cell suspension and cultured at 37°C with 5% CO_2_. Subsequently, the cells were re‐stimulated using O antigens, during which GolgiStop (protein transport inhibitor, BD Biosciences, *USA*) was added to inhibit the secretion of cytokines. Antigen‐specific immune responses were then detected by intracellular cytokine staining and FCM.

### 4.17 Statistical Analysis

All data are presented as the mean ± standard error of the mean (SEM). Statistical analyses were performed using GraphPad Prism (*USA*) by one‐way analysis of variance (ANOVA) or unpaired *t*‐tests. A *p*‐value < 0.05 was considered statistically significant. ^*^Means ANOVA analysis. ^#^Means *t*‐tests.

### 4.18 Software Versions

GraphPad Prism (8.4.3); Image J (1.53a); FlowJo (10.4.0); CytExpert (2.5.0.77); CaseViewer (2.4); NIS (5.21.00).

## Author Contributions

**Peng Liu** conducted experiments, data analysis, and wrote and edited the original manuscript. **Qiang Chen** and **Zhe Zhou** assisted in research and data curation. **Yuekang Xu** refined the language of the article. **Jinyao Li** assisted in the writing and editing of the article and conducted project administration and guidance as well as funding acquisition.

## Funding

This work was support by the Tianshan Talent Training Program (2023TSYCLJ0043), Major science and technology program in Xinjiang Uygur Autonomous Region (2025A03007/2025A03007‐1), National Natural Science Foundation of China (32360185), the Graduate Research Innovation Project of Xinjiang Uygur Autonomous Region (XJ2025G025, XJ2026G024), the Outstanding Doctoral Innovation Project of Xinjiang University (XJU2024BS074, XJDX2025YJS058).

## Conflicts of Interest

The authors declare no conflicts of interest.

## Supporting information




**Supporting File**: advs77089‐sup‐0001‐SuppMat.docx.

## Data Availability

The data that support the findings of this study are available from the corresponding author upon reasonable request.
