# Supplementary material for: PROTAC‐Engineered Protein/DNA Nanoantigen is a Potent Booster for Dendritic Cell Vaccines in Cancer Immunotherapy
Source: Adv Sci (Weinh). 2026 Aug 11;13(61):e77089. doi: 10.1002/advs.77089 (PMC13460275; doi:10.1002/advs.77089)
Supplement: Supplementary file 1 [file ADVS-13-e77089-s001.docx]

**PROTAC-Engineered Protein/DNA Nanoantigen is a Potent Booster for Dendritic Cell Vaccines in Cancer Immunotherapy**

Peng Liu, Qiang Chen, Zhe Zhou, Yuekang Xu, Jinyao Li*

*Xinjiang Key Laboratory of Biological Resources and Genetic Engineering, College of Life Science and Technology, Xinjiang University, Urumqi 830017, China*

*Corresponding author: Jinyao Li ([ljyxju@xju.edu.cn](mailto:ljyxju@xju.edu.cn)).

Contents

1. Optimization results of HOA expression;
2. Primer sequences of PCR or qRT-PCR;
3. Sequencing results of HOA-pET32a, O-pcDNA3.1, and OA-pcDNA3.1;
4. WB verification of plasmid transfection;
5. Transmission electron microscope image;
6. Stability test of HpOAC;
7. Ca^2+^ release in the cytoplasm of DCs by HpOAC;
8. Comparison of the adjuvant effects between soluble HOA or pOA and granular HpOAC;
9. Comparison of O levels after DC treatment with HOA + Lipo-pOA and HpOAC;
10. Ovalbumin (O) IP antibody titration and specific detection;
11. Organ index of tumor-bearing mice;
12. Immune cell typing in the spleen of tumor-bearing mice;
13. Organ index of tumor-bearing mice.
14. Flow cytometry gating scheme

**1. Optimization results of HOA expression**

Prior to purification, the expression conditions for HOA were optimized by varying the IPTG concentration, induction temperature, and induction duration. The results showed that the highest expression level of HOA in the BL21 strain was achieved under induction with 0.5 mM IPTG at 25℃ for 9 hours, which were the conditions ultimately adopted for protein expression.

**Figure S1.** **The SDS-PAGE result diagram of HOA protein expression**.

**2. Primer sequences of PCR or qRT-PCR**

The primers used for PCR and qRT-PCR were synthesized by Sangon Biotech (*China*).

**Table S1 Primer sequences of PCR or qRT-PCR**

| Primer | Sequence (5'-3') |
| --- | --- |
| HOA-F | CACCATCACCATATGGGCTCCATCGGTGCAGCAAGCAT |
| HOA-R | CCGATGGAGCCCATATGGTGATGGTGATGATGCATATG |
| O-F | CTAGCTAGCATGGGCTCCATCGGT |
| O-R | CCGCTCGAGTAAAGGGGAAACACATCTGCCA |
| OA-F | CTAGCTAGCATGGGCTCCATCGGT |
| OA-R | CCGCTCGAGTAATGGAATGTAGGGGGCCAAT |
| GAPDH-F | ACGGCAAATTCAACGGCACAGTCA |
| GAPDH-R | CATTGGGGGTAGGAACACGGAAGG |

**3. Sequencing results of HOA-pET32a, O-pcDNA3.1, and OA-pcDNA3.1**

The obtained plasmids—HOA-pET32a, O-pcDNA3.1, and OA-pcDNA3.1—were subjected to DNA sequencing. The sequencing results were then aligned with the original plasmid sequences, confirming that HOA, O, and OA all possessed the correct DNA sequences.

**Figure S2.** **Plasmid maps and sequencing results.**

**4. WB verification of plasmid transfection**

DCs were transfected with pO or pOA using Lipofectamine 3000. After 72 h, the cells were harvested and lysed, and the intracellular levels of O protein in the cytosol were examined by WB. The results confirmed that both pO and pOA were successfully transfected into DCs and subsequently translated into protein.

**Figure S3.** **Western blot analysis of cytosolic O protein in DCs.**

**5. Transmission electron microscope image**

We prepared HpOAC nano-antigens according to the protocol described in Materials and Methods, and characterized them using transmission electron microscopy (TEM). The TEM images were consistent with the scanning electron microscopy (SEM) results, showing that HpOAC exhibited a spherical morphology with a particle size of approximately 300 nm.

**Figure S4.** **Transmission electron microscope image of HpOAC.**

**6. Stability test of HpOAC**

We dispersed HpOAC in complete RPMI-1640 medium containing 10% serum, and collected HpOAC samples for particle size measurement at 0, 12, 24, and 48 h. At the 48 h time point, two conditions were included: with DCs and without DCs. The results showed that the average particle size of HpOAC was approximately 350±25 nm at all time points, with no obvious aggregation.

**Figure S5.** **Stability test of HpOAC when incubated with DC.**

**7. Ca^2+^ release in the cytoplasm of DCs by HpOAC**

DCs were stimulated with HpOAC for different time points (0, 3, 6, 12, 18, and 24 h), and at each time point, the cytosolic Ca^2+^ concentration was measured using a Ca2+ fluorescent probe (Fluo‑4). The results showed that the Ca^2+^ concentration began to rise at 6 h and reached a peak at 18 h.

**Figure S6.** **Ca^2+^ release kinetics assay.**

**8. Comparison of the adjuvant effects between soluble HOA or pOA and granular HpOAC**

DCs were treated with equivalent doses (50 μg/mL) of HOA protein alone, pOA plasmid alone, HOA+pOA, or HpOAC for 48 h (the time point at which the DC vaccine was prepared for use). The results showed that the levels of DC maturation and XPT in the HpOAC-treated group were substantially higher than those in the groups treated with HOA alone, pOA alone, or HOA+pOA, even though the antigen concentrations in the HOA or pOA groups were higher than that in the HpOAC group.

**Figure S7.** **Comparison of the adjuvant effects of soluble HOA and pOA with particulate HpOAC.**

**9. Comparison of O levels after DC treatment with HOA+Lipo-pOA and HpOAC**

After pretreating DCs with MG132, we treated the cells with either HOA protein plus pOA plasmid transfected using Lipofectamine 3000 or with HpOAC for 48 h. The results showed that the cytosolic O level in the HpOAC-treated group was substantially higher than that in the group treated with HOA+Lipo-pOA.

**Figure S8.** **The level of O after stimulating DC with HOA+Lipo-pOA and HpOAC.**

**10. O IP antibody titration and specific detection**

To determine the optimal amount and specificity of the anti-O antibody for IP, antibody titration and specificity tests were performed. The results showed that 4 μg of antibody did not precipitate more O protein than 2 μg, indicating that 2 μg was sufficient to immunoprecipitate all O protein in the sample. Nevertheless, an excess of 5 μg antibody was used in subsequent IP experiments. Given that the host and isotype of the IP antibody were rabbit and IgG, respectively, rabbit IgG was used as an isotype control in preliminary experiments, and no specific bands were observed.

**Figure S9.** **Titration and specificity detection of the IP antibody of O.**

**11. Organ index of tumor-bearing mice**

Organ photography and organ index of tumor-bearing mice. Organ index = Organ weight / body weight × 1000.

**Figure S10.** **Organ photography and organ index.**

**12. Immune cell typing in the spleen of tumor-bearing mice**

Effects of DC vaccine prepared by HpOAC on T cells (CD3^+^/CD4^+^/CD8^+^), B cells (CD3^-^ CD19^+^), NK cells (CD3^-^ CD19^-^ CD49b^+^), DC (CD11c^+^), and macrophages (CD11b^+^) in spleen of tumor-bearing mice.

**Figure S11.** **Immune cell typing.**

**13. Organ index of tumor-bearing mice**

Organ photography and organ index of tumor-bearing mice. Organ index = Organ weight / body weight × 1000.

**Figure S12.** **Organ photography and organ index.**

**14. Flow cytometry gating scheme**

The gating schemes for all the flow cytometry experiments involved in this study are as follows.

**Figure S13.** **Flow cytometry gating scheme.**
